# Effect of the environment microbiota on the flavour of light-flavour *Baijiu* during spontaneous fermentation

**DOI:** 10.1038/s41598-018-21814-y

**Published:** 2018-02-21

**Authors:** Xiao-Na Pang, Bei-Zhong Han, Xiao-Ning Huang, Xin Zhang, Lin-Feng Hou, Ming Cao, Li-Juan Gao, Guang-Hui Hu, Jing-Yu Chen

**Affiliations:** 1 Beijing Laboratory of Food Quality and Safety, College of Food Science and Nutritional Engineering, China Agricultural University, Beijing, 100083 China; 2 Beijing Advanced Innovation Center for Food Nutrition and Human Health, China Agricultural University, Beijing, 100083 China; 3 Technology Center, Shanxi Xinghuacun Fenjiu Distillery Co. Ltd., Fenyang, 032205 China; 4 Department of Biotechnology, Beijing Center for Physical and Chemical Analysis, Beijing, 100089 China

## Abstract

Light-flavour *Baijiu* is a type of Chinese liquor with a pure and mild flavour produced by traditional spontaneous solid-state fermentation. The flavour of this liquor has been found to vary in the different periods of annual production. To explore the factors affecting flavour, the microbiota of the surrounding environment, starter and fermentation process in different periods were investigated. Results showed that the ester content and acidity of light-flavour *Baijiu* were significantly lower when annual production was resumed after a summer break. HCA plot of volatile flavour profile and bacterial PCoA results indicated that the differences occurred at later stages, mainly due to different structures of *Lactobacillus*. Correlation analysis by O2PLS indicated that *Lactobacillus* positively correlated with esters. Species-level analysis showed that the lack of *L. acetotolerans* on the surface of the jar might cause a lag in fermentation and lower ester content. Thereafter, *L. acetotolerans* was revived during fermentation and enriched on the surface of the jar, which promoted ester formation. As important sources of *L. acetotolerans*, the air and fermentation jars played a critical role during fermentation. Therefore, this systematic study on environmental microbial ecology is valuable for quality control and to explore environmental microbiota functions during spontaneous fermentation.

## Introduction

Over thousands of years, humans have optimized the conditions including temperature, moisture, and salinity to promote the growth of certain microbial communities and obtain various fermented foods such as cheese, alcoholic beverages, sourdough, soy sauce, and vinegar^1,2^. Although most modern fermented foods are inoculated with defined starter cultures, indigenous microorganisms in traditional spontaneous fermentation are often considered to increase the flavour complexity of these foods^3^. A study on the cheese rind microbial community indicated that at least 60% of the bacteria and 25% of the fungi originate from environmental sources^4^. The facility-specific microbiota may play a role in shaping site-specific product characteristics^5^.

*Baijiu*, Chinese liquor, is a type of traditional distilled spirit drink produced by spontaneous mixed-culture solid-state fermentation. The traditional process of *Baijiu* mainly includes material preparation, *Daqu* (starter) preparation, alcoholic fermentation, solid-state distillation, and aging^6^. Because *Daqu* preparation and alcoholic fermentation are carried out under semi-controlled conditions, specific microbiota have been well enriched with the unique ecological environments and manufacturing procedures through repeated practices for a long time^7,8^. The specific microbiota, together with different materials and processing procedures, created various types of *Baijiu*. Based on flavour characteristics, *Baijiu* can be divided into 12 categories^7^. Sauce-flavour *Baijiu*, strong-flavour *Baijiu*, and light-flavour *Baijiu* comprise the three dominating categories. The alcoholic fermentation of light-flavour *Baijiu* is carried out in pottery earthen jars instead of mud pits, which are used for the other two types of *Baijiu*. The importance of fermentation pits and earthen jars has been recognized through practice for thousands of years. Recent research revealed that the microbial community in the pit mud and the grade of pit mud correlated closely with the quality of *Baijiu*^9,10^. *Clostridia* inhabiting the fermentation pit mud have been reported to produce caproic acid and ethyl caproate, which have been identified as key flavour substances of strong-flavour *Baijiu*^11^. Research also illustrated that the microorganisms from air are involved in the fermentation process^12^.

As spontaneous fermentation, *Baijiu* production is largely influenced by environmental factors, especially in the summer. There is a production break during July and August due to a decline in the productivity and quality of light-flavour *Baijiu*. The annual production is divided into three periods: (1) *Lipai* period (LP) refers to the beginning of a production cycle in September after the summer production break, (2) *Tiaopai* period (TP) refers to the last period of production before production break in the summer, (3) *Yuanpai* period (YP) refers to the production period between LP and TP. Throughout the annual production, it was found in common that the ester content in light-flavour *Baijiu* produced during LP was found to be significantly lower than that produced during YP and TP, although the same *Daqu* was used. Esters are an important group of volatile compounds that contribute to the flavour of *Baijiu* and are regarded as a quality index. The major flavour compounds of light-flavour *Baijiu* are ethyl acetate balanced with ethyl lactate, which are represented by *Fenjiu*^13^. Environmental microorganism from fermenters, tools, staffs, and air in the material preparing room are thought to cause a difference to the flavour and quality during LP.

Previous studies mainly focused on the microbiota dynamics during *Daqu* and *Baijiu* fermentation^14,15^; however, information about the environmental microbiota is still fragmented. The advent of high-throughput sequencing (HTS) technologies has enabled us to investigate microbial communities in the air, contact surfaces, and fermented products^16^. In this study, we attempted to provide an insight into the composition and dynamics of environmental microbial communities present in different alcoholic fermentation periods. Multivariate statistical analyses were applied to improve the understanding on the effects of the environmental microbiota on the quality of *Baijiu* quality. These findings should be immensely helpful for exploring environmental microbiota functions during spontaneous fermentation.

## Results

### Dynamics of physicochemical characteristics in different fermentation periods

**Figure 1:**
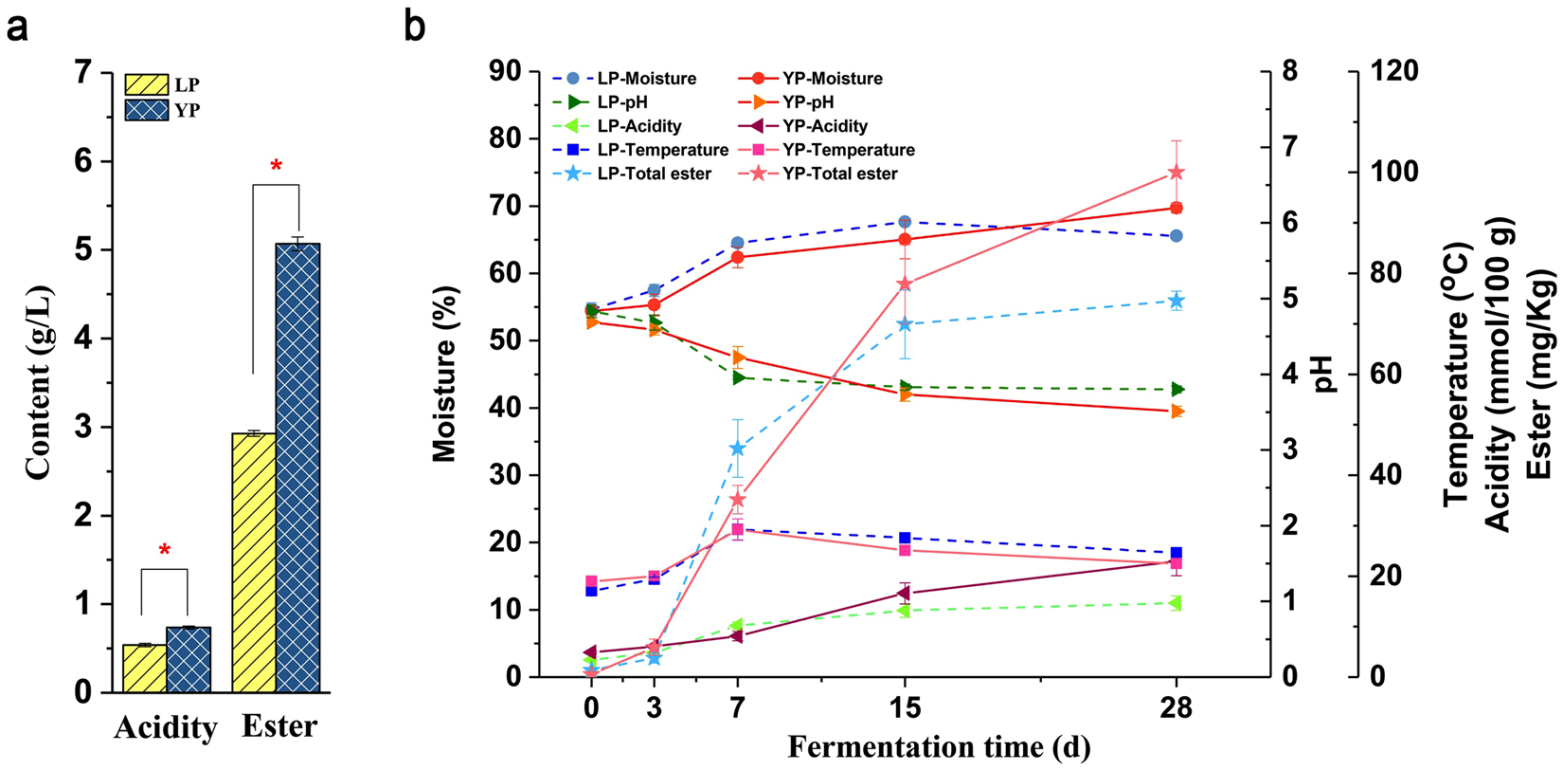
Dynamics of physicochemical characteristics during light-flavour *Baijiu* fermentation in different periods. (**a**) Comparison of total acid and ester in light-flavour *Baijiu* in LP and YP. (**b**) Physicochemical characteristics dynamics during light-flavour *Baijiu* fermentation in LP and YP. LP: *Lipai* period; YP: *Yuanpai* period. ^*^There are significant differences between LP and YP.

As shown in Fig. 1a, the ester content and acidity of *Baijiu* during LP and YP were significantly different. The ester content in *Baijiu* during LP was about 2.93 g/L, which was significantly lower than 5.07 g/L in YP (*p* < 0.01). The acidity of *Baijiu* during LP was 0.54 g/L which was lower than 0.74 g/L in YP (*p* < 0.01).

The dynamics of the physicochemical characteristics throughout the light-flavour *Baijiu* fermentation during LP and YP are shown in Fig. 1b. Temperature changes in the core of earthen jars during fermentation were continuously monitored. The dynamic trend during LP and YP were similar, the core temperature at the beginning of fermentation was around 17 °C and 18 °C. As fermentation progressed, the core temperature slowly increased to 30 °C after 7 days and was maintained around 30 °C for 3 to 4 days; then it decreased gradually to 22–24 °C at the end of the fermentation process. The moisture content at the beginning was about 54.5% and significantly increased to 66.4% on day 15 during both LP and YP. However, the moisture content declined to 65.6% during LP, which is lower than 69.7% observed at the end of YP. The pH declined steadily to 3.80 and the titratable acidity increased gradually to 14.90 mmol/100 g on day 15 during both LP and YP. Afterward, pH and titratable acidity was maintained at the end of LP, but the pH decreased to 3.51 and the acidity increased to 22.90 mmol/100 g at the end of YP fermentation. The ester content increased rapidly from day 3 to 15, and was maintained around 74.59 mg/Kg at the end of LP, whereas ester content continually increased to 100.04 mg/Kg at the end of YP. Generally, the evolution trends of the physicochemical characteristics were similar during LP and YP. However, the moisture, acidity, and ester content at the end of YP were significantly higher than that during LP (*P* < 0.05).

### Multivariate analysis of volatile flavours during different fermentation periods

**Figure 2:**
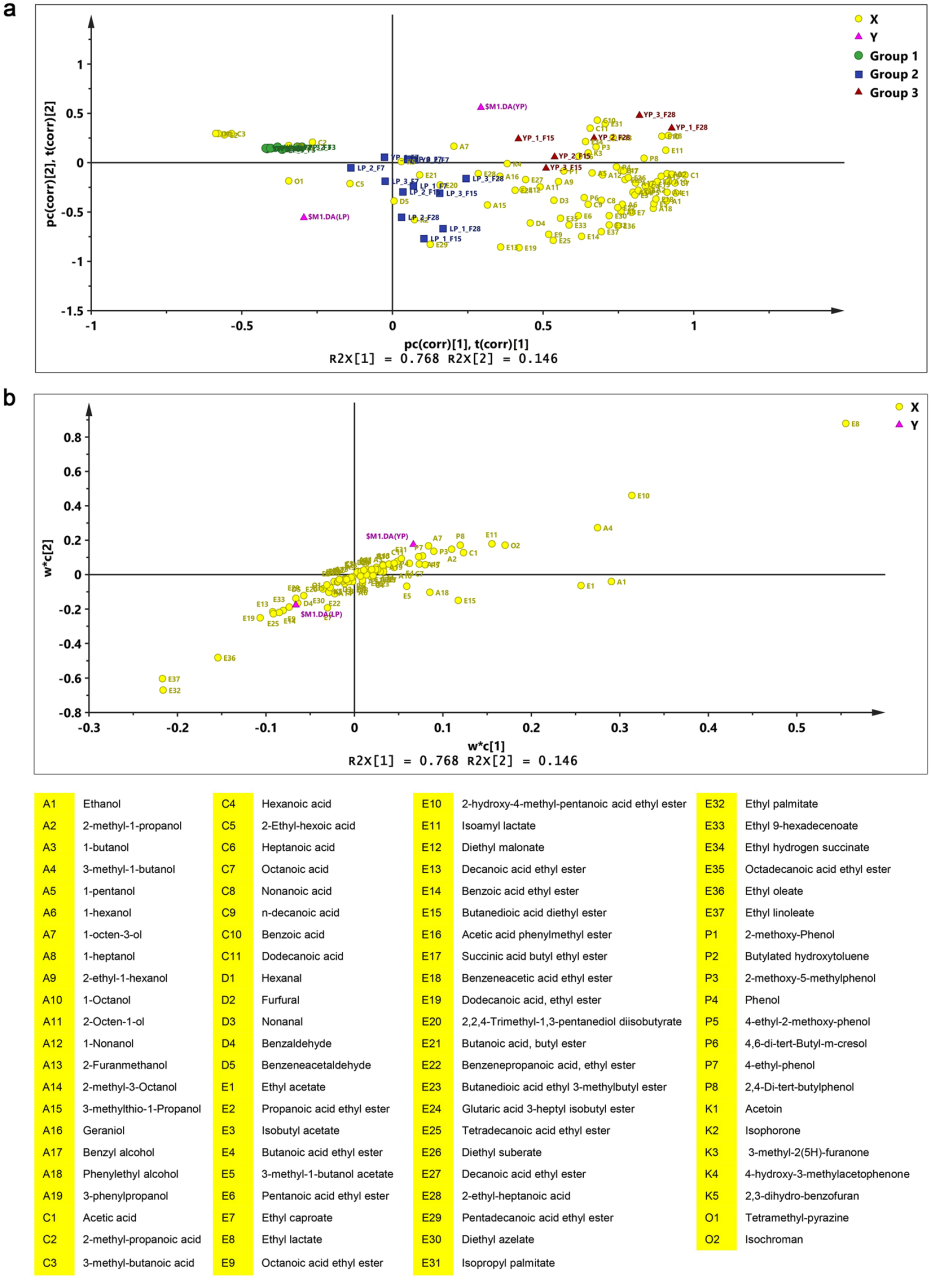
PLS-DA analysis of volatile flavours during light-flavour *Baijiu* fermentation in different periods. (**a**) Biplot superimposed the scores and loadings of PLS-DA. (**b**) Loading plot of PLS1 and PLS2. LP: *Lipai* period; YP: *Yuanpai* period. The middle number in the sample code represents different plants. F followed by a number means fermentation time. X: yellow dots means volatile flavour. Y: pink triangle defines the different classes membership of LP or YP in PLS-DA model.

A total of 87 volatile flavours were detected during light-flavour *Baijiu* fermentation, namely, 37 esters, 19 alcohols, 11 organic acids, 5 ketones, 5 aldehydes, 8 phenols, and 2 others (Fig. 2). Hierarchical cluster analysis (HCA) and partial least square discriminate analysis (PLS-DA) were carried out to summarize volatile flavour data. HCA plot (Fig. S1) revealed the fermentation during YP could be divided into 3 stages based on volatile flavours profiles: stage 1, days 0 and 3; stage 2, day 7; stage 3, day 15 and 28. The volatile profiles on days 0 and 3 during LP were similar to YP, whereas the samples of days 15 and 28 were in the same group as the samples from day 7 in YP (Fig. 2a). PLS-DA was performed to sharpen the separation between samples of LP and YP. As shown in Fig. 2a, the results explained 91.4% of the total variance with R2X (76.8%) and R2Y (14.6%). Three groups could be clearly defined according to the clusters retrieved from the HCA. The categories and the content of volatile compounds increased with fermentation (Fig. 2a). The detailed distinctions of volatile profiles in different periods were revealed by the loading plot of PLS-DA (Fig. 2b). Terms with large VIP, larger than 1, are the most relevant for explaining Y. The VIP of ethyl acetate, ethanol, ethyl lactate, butanedioic acid diethyl ester, 3-methyl-1-butanol, phenylethyl alcohol, 2-hydroxy-4-methyl-pentanoic acid ethyl ester, 3-methyl-1-butanol acetate, and isochroman which larger than 1.0 and showed strong correlation with YP. Ethyl palmitate, ethyl linoleate, ethyl oleate, ethyl caproate, and 2,2,4-trimethyl-1,3-pentanediol diisobutyrate (VIP > 1.0) contributed to the specificity of LP. Detail VIP value was provided in the supplemental Dataset S1. Ethyl acetate and ethyl lactate act as the main esters in light-flavour *Baijiu*; the contents during YP were significantly higher than those during LP, which is consistent with the results shown in Fig. 1b. HCA clustering and PLS-DA classification highlighted that the volatile flavour profiles were strongly affected by the fermentation time and period.

### Microbial communities of the environment and starter in different fermentation periods

**Figure 3:**
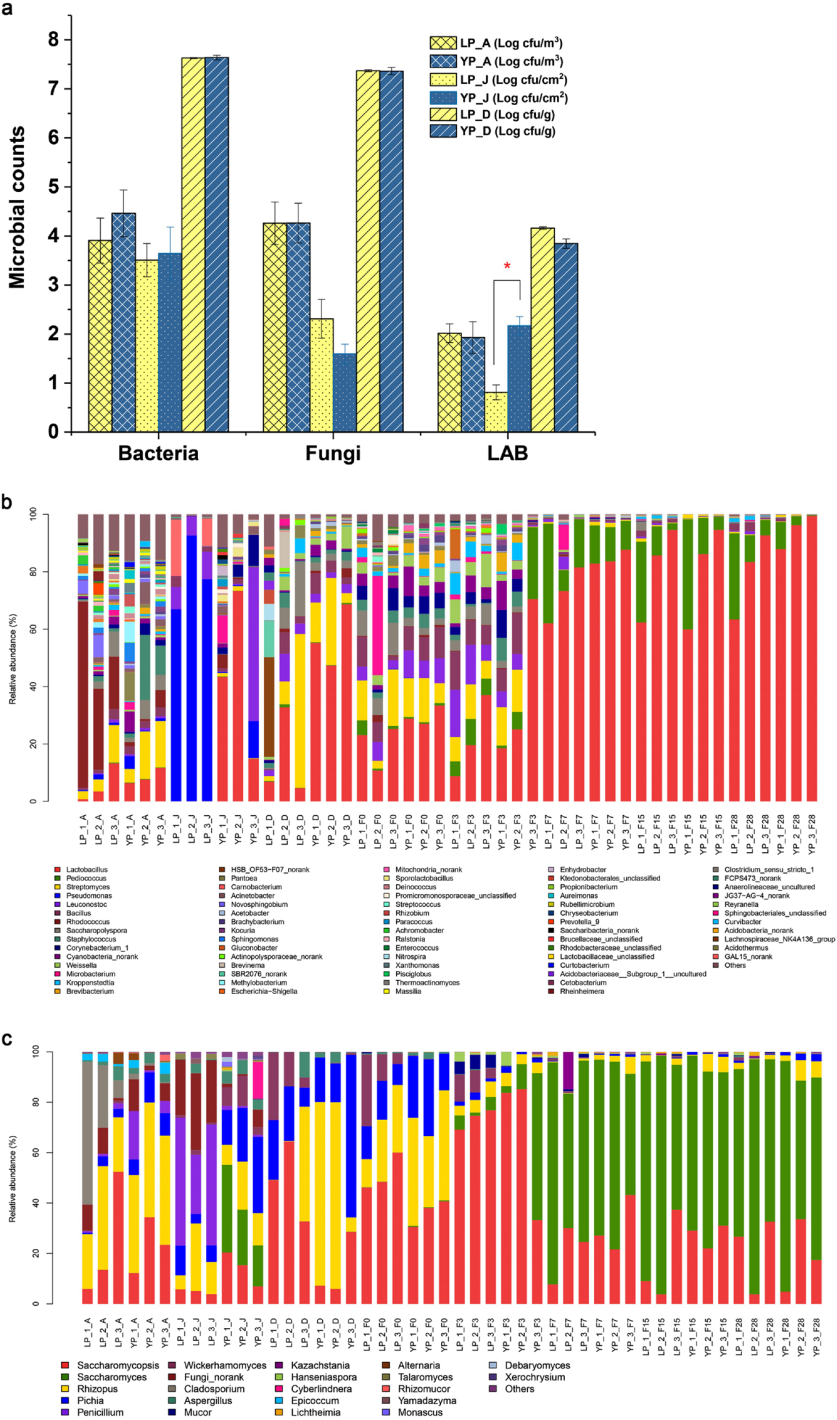
Environmental microbiota and dynamics during light-flavour *Baijiu* fermentation of different periods at the genus level. (**a**) Viable microbial counts of of air, surface of the jar, *Daqu*. (**b**) Bacterial structure of air, surface of the jar, *Daqu*, and fermented grains at the genus level. (**c**) Fungi structure of air, surface of the jar, *Daqu*, and fermented grains at the genus level. The genera with less than 1% were grouped into others. LP: *Lipai* period; YP: *Yuanpai* period; A: air; J: surface of the jar; D: *Daqu*; F followed by a number means fermentation time.

As an important source of microorganisms contributing to fermentation, the microbial communities of the air of the material processing room, the inner surface of the jar, and *Daqu* were detected by both a culture-dependent and an amplicon sequencing method as shown in Fig. 3. For microbes in the air and *Daqu*, there were no significant differences between the two production periods according to the result of viable microbial counts (Fig. 3a). The average counts of the two periods for bacteria, fungi, and lactic acid bacteria (LAB) in the air were 4.2, 4.3, and 2.0 log CFU/m^3^, respectively. The average counts of the two periods for bacteria, fungi, and LAB in *Daqu* were 7.6, 7.4, and 4.0 log CFU/g, respectively. For microbes on the surface of earthen jars, the average counts of the two periods for bacteria and fungi were 3.6 and 1.9 log CFU/cm^2^, respectively. In the case of LAB, it was 2.2 log CFU/cm^2^ in YP and 0.8 log CFU/cm^2^ in LP, which were significant different (*P* < 0.01).

**Figure 4:**
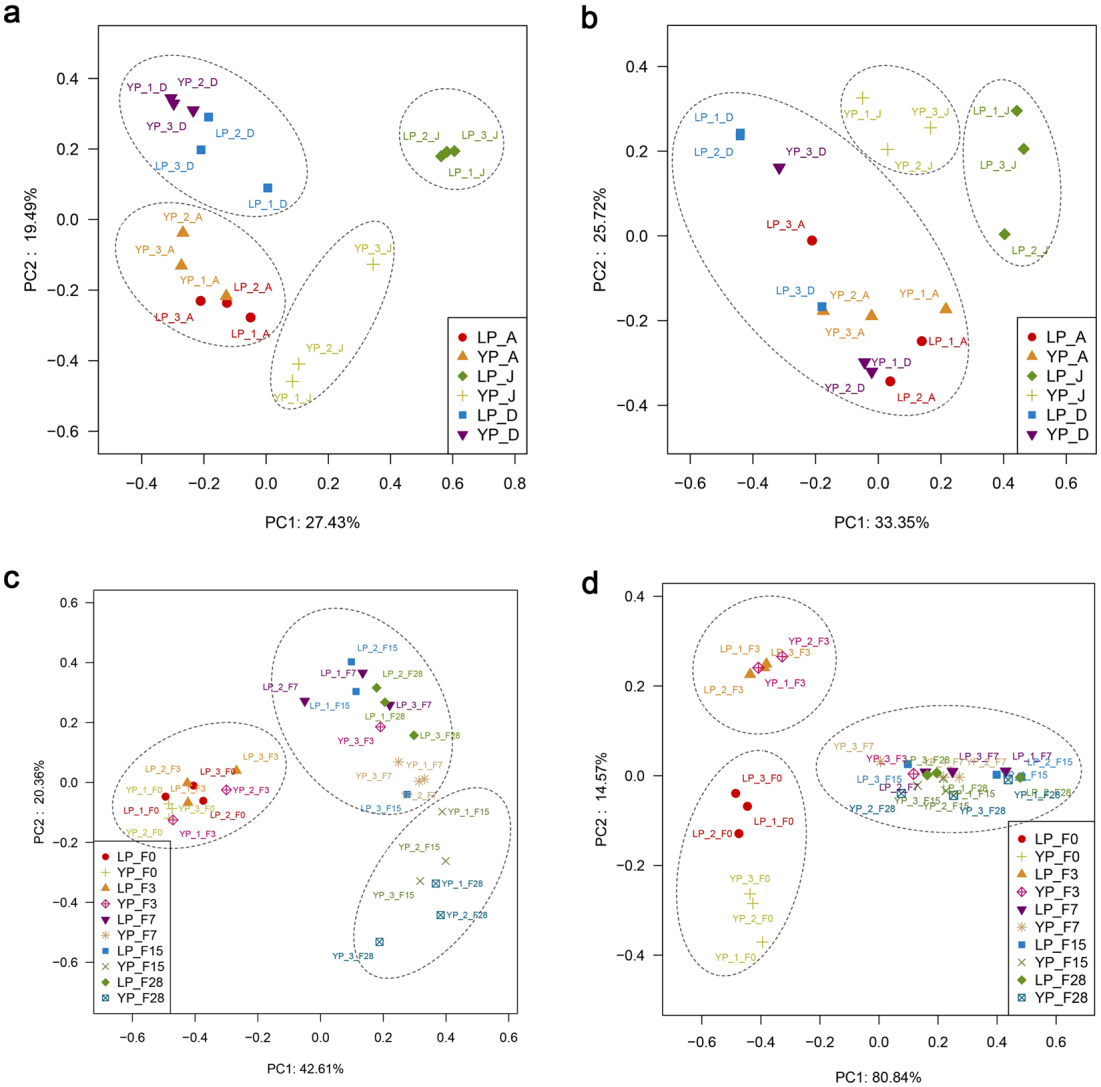
PCoA analysis of microbiota during light-flavour *Baijiu* fermentation at the OTU level based on Bray-Curtis distance. (**a**) PCoA of bacterial community from air, surface of the jar, and *Daqu*. (**b**) PCoA of fungal communities from air, surface of the jar, and *Daqu*. (**c**) PCoA of bacterial communities during fermentation. (**d**) PCoA of fungal communities during fermentation. LP: *Lipai* period; YP: *Yuanpai* period; A: air; J: surface of the jar; D: *Daqu*. F followed by a number means fermentation time.

To better reveal the differences between the microbial ecosystems during the two periods, HTS approach was used to explore the microbial communities in the environment, starter, and fermentation processes. Detailed sequencing information is provided in the supplemental Dataset S1. As for bacteria, *Lactobacillus* dominated on the surface of the jar during YP with an average abundance of 43.8%, followed by *Leuconostoc* (18.1%). In contrast, *Pseudomonas* was in high abundance, up to 79.0% on the surface of the jar during LP, followed by *Carnobacterium* (9.6%) and *Leuconostoc* (8.0%), whereas *Lactobacillus* was at lower level (0.02%) as shown in Fig. 3b. *Pseudomonas* and *Carnobacterium* were mainly found on the surface of the jar during LP. *Lactobacillus* and *Leuconostoc* predominated on the surface of the jar during YP and were associated to the fermentation process. To reveal the similarities and dissimilarities of microbial communities, groups were retrieved from result of PCoA as shown in Fig. 4. As shown by the PCoA plot (Fig. 4a), the samples from the surface of the jar during LP were clearly differentiated from YP based at the OTU (operational taxonomic unit) levels. The PCoA result was consistent with the results detected by the culture-dependent method. Both in LP and YP, *Rhodococcus*, *Streptomyces*, and *Lactobacillus* predominated in the air, followed by *Novosphingobium*, *Saccharopolyspora*, *Staphylococcus*, *Pantoea*, and *Acinetobacter*, while *Cyanobacteria_*norank represented 2.4% of the total bacterial population in the air (Fig. 3b). The PCoA results indicated a similar bacterial structure in the air during the two periods (Fig. 4a). *Daqu* was dominated by *Lactobacillus*, *Streptomyces*, *Saccharopolyspora*, *Bacillus*, and *Leuconostoc*. PCoA results revealed similar bacterial structures in *Daqu* during the two periods (Fig. 4a). Moreover, analysis of Similarity (ANOSIM) was used to analyse the significance of groups for microbial community retrieved from PCoA. The results of ANOSIM revealed statistically significant differences among the groups of microbial community (*p* < 0.05) which were presented in Table S2. A joint analysis of the results of ANOSIM and PCoA indicates that the similarity and difference of microbial communities.

As for Fungi, PCoA results showed similar trends with bacteria; the samples from the surface of the jars could be divided into two groups, and there were no obvious clusters dividing air and *Daqu* samples from the different periods (Fig. 4b). *Penicillium* predominated on the surface of the jar during LP, followed by *Rhizopus*, *Pichia*, and *Saccharomycopsis*, while unaffiliated fungi represented 25.7% of the total fungal population (Fig. 3b). *Saccharomyces* was in very low abundance, below 0.1%. In contrast, *Saccharomyces* predominated on the surface of the jar during YP with an average abundance of 24.4%, followed by *Pichia*, *Saccharomycopsis*, and *Rhizopus*. In the air of the preparing room, *Rhizopus, Saccharomycopsis*, and *Pichia* were also the predominant genera. *Cladosporium* was in a higher abundance during LP, but was not found in the fermented grains. *Rhizopus*, *Saccharomycopsis*, *Pichia*, and *Wickerhamomyces* had an abundance of above 90% in *Daqu* during both LP and YP; the average abundance of *Saccharomyces* in *Daqu* was below 0.1%. Because *Saccharomyces* was the predominant genus during the fermentation process after 7 days in LP and YP, *Daqu* and surface of the jar could be an important source of *Saccharomyces* (Fig. 3b).

### Microbial community dynamics during fermentation processing

*Lactobacillus*, *Streptomyces*, *Saccharopolyspora*, *Bacillus*, *Leuconostoc*, *Microbacterium*, and *Weissella* were the prevailing bacterial genera on days 0 and 3, with a minor presence of *Pediococcus* (Fig. 3a). After 7 days, *Lactobacillus* and *Pediococcus* became the predominant genera during both LP and YP (Fig. 3b). *Saccharomycopsis*, *Rhizopus*, and *Pichia* were the predominant fungal genera on day 0. As fermentation progressed, *Rhizopus* and *Pichia* decreased, while *Saccharomyces* increased gradually and became the most prominent genus after 7 days (Fig. 3b). Both bacteria and fungi structures were similar during fermentation in the two production periods at the genus level. Fungal PCoA (Fig. 4d) results also revealed a similar structure and dynamics between the two periods. Bacterial PCoA (Fig. 4b), based on the Bray-Curtis distance at OTU level, revealed that there were some differences between the two periods. The samples from YP were separated into 3 groups: I, days 0 and 3; II, day 7; and III, days 15 to 28. Samples of days 0 and 3 from the two periods were similar, but the samples from days 15 and 28 were in different clusters. The samples on day 15 and 28 in LP were clustered together with the samples of day 7. The divided groups were consistent with PLS-DA and HCA results of volatile flavour profiles, which indicate the correlation between microorganisms and volatile flavours.

**Figure 5:**
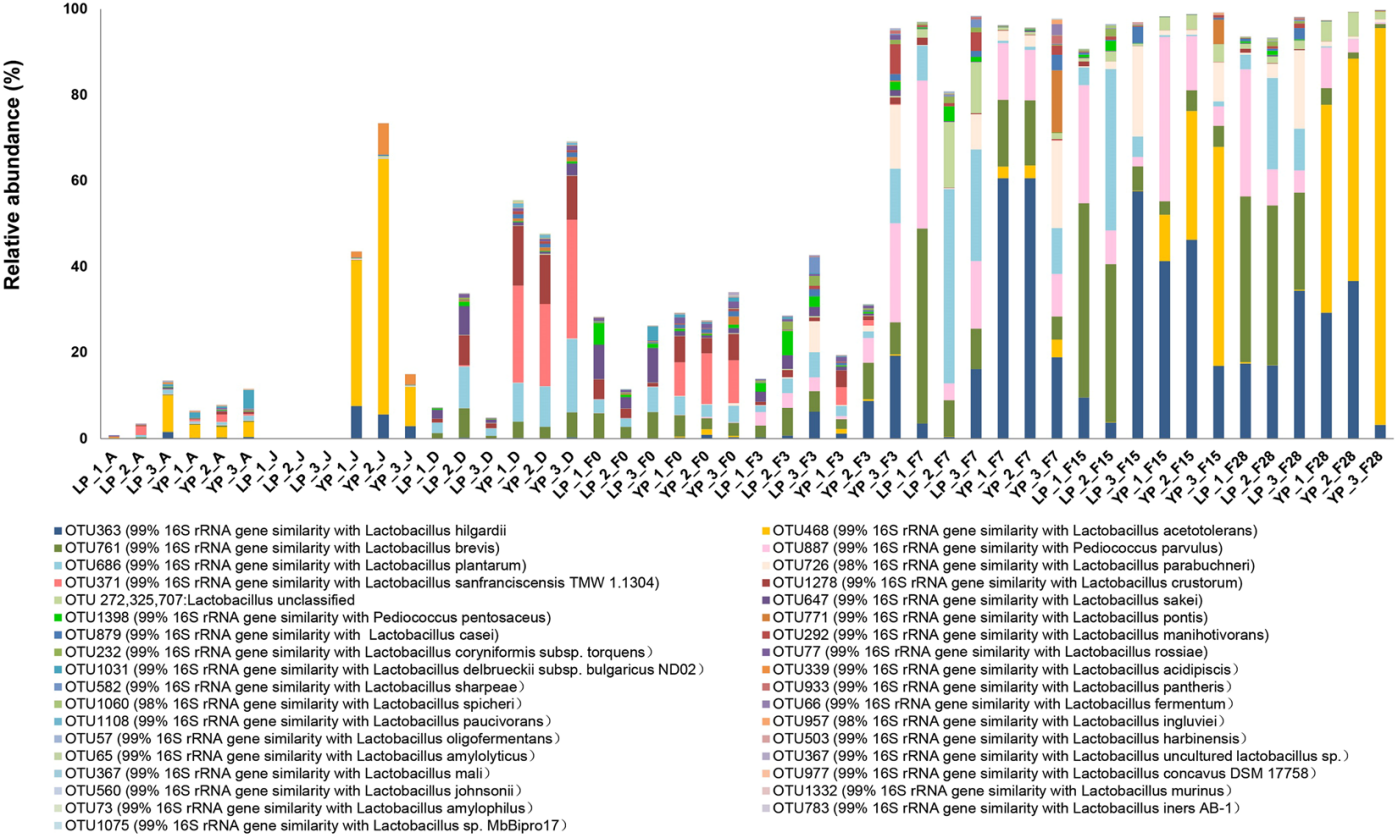
*Lactobacillus* and *Pediococcus* dynamics during light-flavour *Baijiu* fermentation at different periods based on the change of percentages of OTU belongs to the two genera in the samples as revealed by HTS.

To further estimate the composition of bacterial communities at species level, specific analyses were carried out to investigate the dominant genus, *Lactobacillus* and *Pediococcus*, using the relative abundance of OTUs as shown in Fig. 5. Taxonomic details up to species level are reported in Dastaset S1. At the beginning of fermentation in LP, the average percentage of *Lactobacillus* was about 19.7%, the most abundant OTUs were *Lactobacillus brevis, Lactobacillus plantarum*, *Lactobacillus crustorum*, and *Lactobacillus sakei*, which mainly came from *Daqu*. The average percentage of *Pediococcus* was 2.3%, which was composed of OTUs of *Pediococcus parvulus* and *Pediococcus pentosaceus*. The composition of *Lactobacillus* and *Pediococcus* did not vary greatly from day 0 to day 3. However, on day 7, the proportion of *Lactobacillus* and *Pediococcus* rapidly increased to 72.3% and 19.7%, respectively, and the OTUs were mainly *L. plantarum* (26.5%), *L. brevis* (21.1%), and *P. parvulus* (18.1%). At a later stage, OTU of *L. plantarum* declined to 11.5% on day 28 of LP. Additionally, OTUs of *L. brevis* (32.8%), *Lactobacillus hilgardii* (22.9%), and *P. parvulus* (14.4%) were the predominant bacterial species on day 28 of LP.

The bacterial composition on day 0 and 3 of YP was similar to LP. The average abundance of *Lactobacillus* was about 29.6% on day 0. The only difference was the content of OTU of *Lactobacillus sanfranciscensis* (9.9%) in YP, which mainly came from *Daqu*. On day 7, OTU of *L. sanfranciscensis* declined to 0.1%, and OTU of *L. hilgardii* rapidly increased to 46.7%, followed by *L. brevis* (12.0%) and *P. parvulus* (11.6%). As the fermentation progressed, OTU of *L. hilgardii* (23.0%), OTUs of *L. brevis* (2.1%), and *P. parvulus* (4.3%) declined sharply. Additionally, OTU of *L. acetotolerans* became the most predominant species (64.3%) on day 28 of YP. However, in LP, OTU of *L. acetotolerans* was at levels as low as 0.2%. In general, as the most abundant bacteria at a later stage in YP, OTU of *L. acetotolerans* caused a significant difference in bacterial composition between LP and YP. OTU of *L. acetotolerans* was hardly detected in *Daqu*, the air, or day 0 samples, but a higher proportion was found on the surface of the jar. From this, it was determined that the surface of the jar was the main source of OTU of *L. acetotolerans*. This might be responsible for the observed differences between the two periods.

### Correlation between microbiota and volatile flavours during light-flavour *Baijiu* fermentation

**Figure 6:**
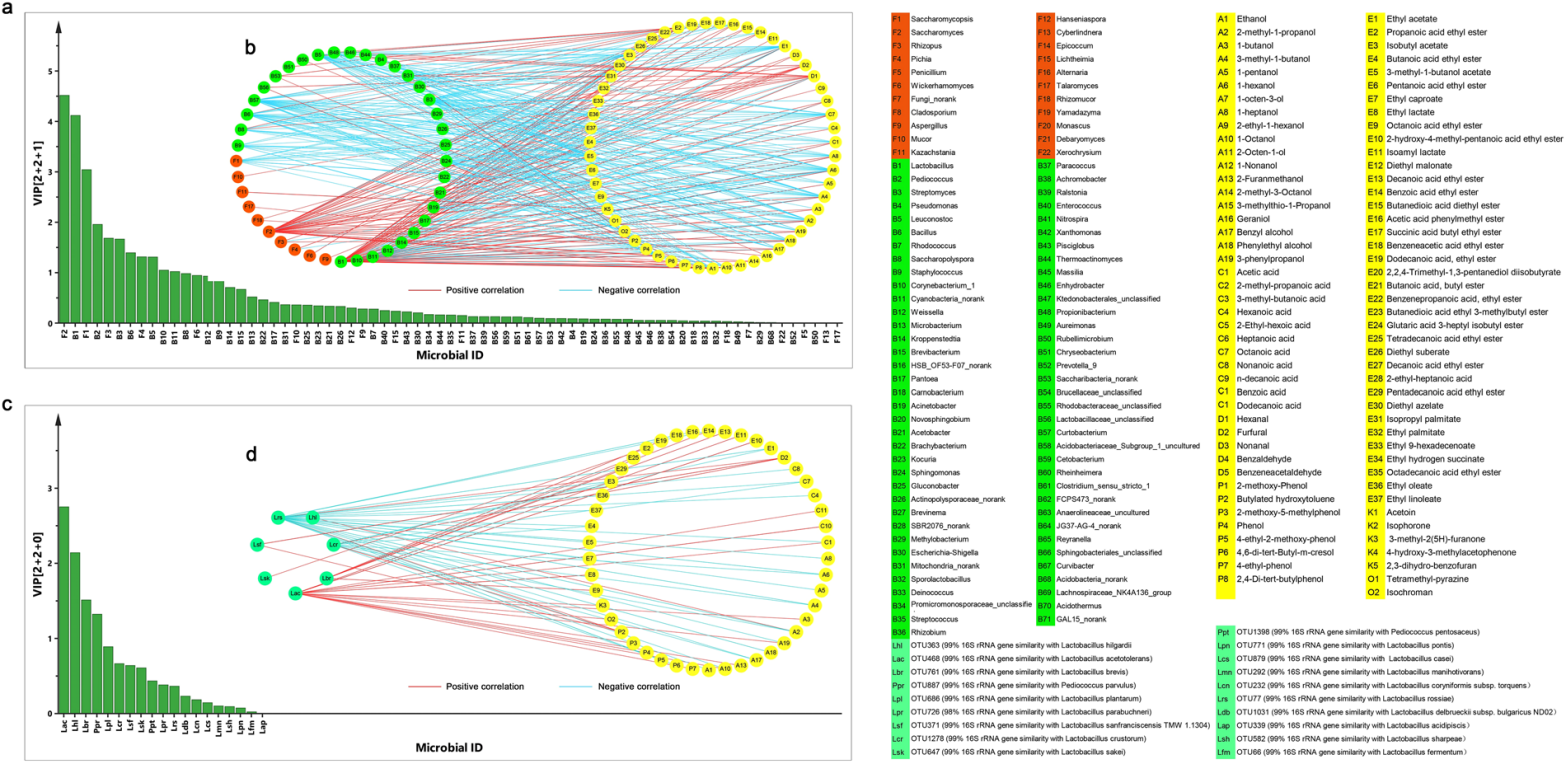
Correlation of microbiota and flavours by O2PLS during light-flavour *Baijiu* fermentation (**a**) VIP plot of microbiota genera. (**b**) The correlated network between microbial genera and flavours during fermentation (|*ρ*| ≥ 0.7). The left side circles represent bacteria (green nodes) and fungi (orange nodes). The right-side circles represent flavours. (**c**) VIP plot of *Lactobacillus* and *Pediococcus*. (**d**) The correlated network between *Lactobacillus* and flavours during fermentation (|*ρ*| ≥ 0.7). The left side nodes represent *Lactobacillus*. The right-side nodes represent the flavours. VIP: Variable importance in the projection.

O2PLS analysis was carried out to analyse the correlation between microbiota and flavours during fermentation. A total of 93 genera including 61 bacteria and 22 fungi were analysed as *X*, and 87 flavours were analysed as *Y*. It was shown that *R*^2^*X(cum)* and *R*^2^*Y(cum)* was 0.854 and 0.936, respectively, and *R*^2^*(cum)* and *Q*^2^*(cum)* of the model was 0.678 and 0.646, respectively (Dataset S1). When the values of *R*^2^ and *Q*^2^ are >0.5, the model is considered to be successful^17^; and the permutation plot strongly indicates that the model is valid (Fig. S2). this suggested that the O2PLS method was well fitted for analysis and prediction. There were 4 fungal vectors and 8 bacterial vectors; a value of VIP > 1.0 indicated important effects on the flavours. *Saccharomyces*, *Saccharomycopsis*, *Rhizopus*, and *Pichia* were the four most important fungal vectors that contributed to the development of flavour during fermentation. There were 7 bacterial genera with VIP value larger than 1.0 which included *Lactobacillus*, *Pediococcus*, *Streptomyces*, *Bacillus*, *Leuconostoc*, *Microbacterium*, and *Corynebacterium*, while *Cyanobacteria*_norank also has a VIP value > 1.0 which indicated that the 8 bacterial vectors may have important effect on the flavours. The correlation coefficient between microbiota and flavours is shown in Dataset S1. The highly correlated part (|*ρ*| ≥ 0.7) with *p*-value < 0.01 was visualised via Cytoscape. As shown in Fig. 6, 31 bacterial genera (green circles in left) and 10 fungal genera (orange circles in left) were highly correlated (|*ρ*| ≥ 0.7) with flavours (yellow circles in right). All the 4 fungal genera (VIP > 1.0) showed a strong correlation (|*ρ*| ≥ 0.7) with flavours. *Saccharomyces* were positively correlated with 39 flavours, including 19 esters in light-flavour *Baijiu* fermentation. *Saccharomycopsis* was negatively correlated with most of flavours. Among the 8 bacterial vectors VIP > 1.0, *Lactobacillus* was highly positively correlated with 41 flavours, including 18 esters. *Streptomyces*, *Bacillus*, and *Leuconostoc* showed a negative correlation with most of flavours (Fig. 6b). The correlation coefficient between *Saccharomyces* and ethyl acetate was 0.9, and the correlation coefficient with ethyl lactate was 0.5. The correlation coefficient between *Lactobacillus* and ethyl acetate was 0.9, and the correlation coefficient with ethyl lactate was 0.6 (Dataset S1). In summary, *Saccharomyces* and *Lactobacillus* were positively correlated with ethyl acetate and ethyl lactate.

A significant difference was found at the bacterial species-level in different fermentation periods (Figs 4, 5); therefore, *Lactobacillus* and *Pediococcus* (as predominant genus with VIP > 1.0) were analysed to explore the correlation between species and flavours using O2PLS. A total of 19 species with an abundance above 1% were analysed as *X*; 87 flavours were analysed as *Y*. *R*^2^*(cum)* and *Q*^2^*(cum)* of the model were 0.816 and 0.713, respectively (Dataset S1), indicating that the O2PLS method was well-fitted for analysis and prediction. Four vectors, OTUs of *L. acetotolerans*, *L. hilgardii*, *L. brevis*, and *P. parvulus* (VIP > 1.0), showed an important effect on flavours (Fig. 6c). OTU of *L. acetotolerans* was highly positively correlated with 15 flavours. The correlation coefficient between OTU of *L. acetotolerans* and ethyl acetate was 0.6, and the correlation coefficient with ethyl lactate was 0.9. OTU of *L. brevis* highly positively correlated with 7 esters, mainly those with long carbon chain. OTU of *L. hilgardii* highly positively correlated with 2 alcohols. OTU of *L. rossiae* showed a negative correlation with most flavours, and other species showed a moderate correlation with flavours (0.04 ≤ |*ρ*| ≤ 0.7). Details of the correlation between microbiota and flavours are listed in (Dataset S1). In general, OTU of *L. acetotolerans* had a moderate or high positive correlation with short chain carbon esters, whereas OTU of *L. brevis* highly positively correlated with 7 esters, mainly those with long carbon chains.

## Discussion

Flavour, as an important quality characteristic of *Baijiu*, has been widely investigated^18,19^. To illustrate the contribution of microorganisms to flavour, bacterial and fungal diversities in *Daqu* and light-flavour *Baijiu* fermentation processes were primarily detected both by culture-dependent and culture-independent methods^20,21^. Specific microbiota have been well enriched with unique ecological environments and manufacturing procedures through repeated practices. However, little attention has been paid to the effect of environment-specific microbiota on the fermentation and characteristics of *Baijiu*. This study aimed to investigate the microbial ecosystems in the environments and dynamics during different fermentation periods to evaluate the effect of environmental microbiota on the flavour of light-flavour *Baijiu*.

Results showed that the general evolution trends of the physicochemical characteristics during fermentation were in line with previous studies^22^. However, the moisture, acidity, and ester content at the end of LP were significantly lower than that of YP (P < 0.05), which resulted in significantly lower acidity and ester content in light-flavour *Baijiu* during LP. Volatile flavour dynamics during fermentation were investigated by HS-SPME-GC-MS. A total of 87 compounds including 37 esters were detected. HCA and PLS-DA of volatile flavours showed that at early stages (days 0 and 3), the samples of LP and YP were in the same group, whereas the samples of later stages in LP (days 15 and 28) were in the same cluster with day 7. The loading plot of PLS-DA revealed that ethyl acetate and ethyl lactate are the most important esters of light-flavour *Baijiu*^23^; however, the content during LP was significant lower than that during YP.

To investigate the cause of this difference, microbial community dynamics were explored by HTS. *Lactobacillus* and *Pediococcus* increased significantly and constituted above 90% of the bacterial composition after day 7 during fermentation (Fig. 3b). Acidity during fermentation highly positively correlated with *Lactobacillus*, which produced lactic acid as the major end product in carbohydrate fermentation and resulted in lower pH. The higher acidity and lower pH tends to reduce overall diversity and change the composition of microbial communities^24^. As for fungal communities, it was found that *Saccharomycopsis*, *Rhizopus*, and *Pichia* were the predominant genera at early stages. While *Rhizopus* and *Pichia* declined with fermentation, *Saccharomyces* increased gradually and became the predominant genus after day 7 in both LP and YP. No obvious differences were found in the fungal composition according to dynamics during fermentation and PCoA results. *Lactobacillus* and *Saccharomyces* as the predominant genera of bacteria and fungi showed strong positive correlation with most volatile flavours during light-flavour *Baijiu* fermentation according to O2PLS analysis results.

The general trends of microbial dynamics during LP and YP were similar. However, PCoA results of bacteria at OTU level indicated differences at later stage during LP and YP which consistent with the results of the volatile flavour profile. Similarly, different species of the same genus can occur in turns during fermentation of cheese or during storage of beef^25,26^. In such cases, an HTS study at the genus level is not informative, and species-level identification is needed to obtain useful information in food^27^. Species-level analysis was carried out in such cases and results showed that *Lactobacillus* composition during LP and YP was apparently different at species level at a later stage. The difference was produced from the OTU of *L. acetotolerans*, which dominated at the later stage in YP, consistent with results of previous studies^15,28^, whereas OTU of *L. acetotolerans* was just at a very low abundance in LP. Furthermore, a correlation analysis revealed that OTU of *L. acetotolerans* was positively correlated with ester lactate and ester acetate. The lack of *L. acetotolerans* in LP may the important factor contributing to the lag in fermentation and lower ester content.

*L. acetotolerans* species enriched during later stages of fermentation, indicating its tolerance to high ethanol concentration and low pH^29^. *L. acetotolerans* has been reported to dominate not only during the fermentation of light-flavour *Baijiu*, but also in strong-flavour and sesame-flavour *Baijiu*^15,21^. However, it was interesting that *L. acetotolerans* was not found in different types of *Daqu*^30,31^ or at the beginning of fermentation. After day 7, it became the most abundant species^15^. During processing and long-time storage of *Daqu*, *L. acetotolerans* was only occasionally detected at a lower abundance in one sample among a large number of samples^32^. In this study, among 6 *Daqu* samples, *L. acetotolerans* was only found in one sample at low abundance (<0.1%). The distribution of *L. acetotolerans* in *Daqu* was consistent with previous studies. Furthermore, the environmental microbial ecosystem was explored and an important finding was that *L. acetotolerans* was found in the samples of air and surface of the jar, especially in high abundance on the surface of the jar in YP; this might play a key role in the fermentation. The presence of *L. acetotolerans* in spontaneous fermentation of sourdough, fermented pickles, and fishes proved its potential occurrence in a relative fermentation environment^33,34^. The distribution characteristic of *L. acetotolerans* on the surface of the jar in different period raises two questions: which technological parameters inhibited their survival or growth during LP, and how did they colonize on the surface and enriched during fermentation. It was supposed that after production breaks the surface of the jar is not suitable for anaerobic *Lactobacillus*, but suitable for aerobic *Pseudomonas*, which predominated on the surface of the jar. Whereas *Pseudomonas* was inhibited due to condition changed with alcoholic fermentation. A study reported that *Lactobacillus* strains isolated from sorghum-based fermented products showed inhibition of *Pseudomonas* biofilm formation^35^. *L. acetotolerans* is reportedly hard to isolate from spoilage beer because of its viable putative non-culturable (VPNC) state in the beer^36^. It was supposed that *L. acetotolerans* might be in a VPNC state during production break in the summer. During LP, *L. acetotolerans* resuscitation with survival situation occurred during fermentation, and was enriched in the air and the surface of the jar; high acetic acid concentrations in YP may have facilitated its growth. This is a topic that needs to be further explored.

Esterase activity has been reported for LAB associated with wine or cheese ripening, such as *Oenococcus*, *Pediococcus*, and *Lactobacillus*^37^. Although the role of LAB in ester formation during *Baijiu* fermentation has been recognized, the effect of LAB is usually controlled because too much ethyl lactate will bring a undesirable flavour in light-flavour flavour *Baijiu*^38^. This study showed that *Lactobacillus* was positively correlated with important esters *in Baijiu*. It was indicated that the lack of *L. acetotolerans* in LP might be an important factor contributing to the lag of fermentation and lower ester content.

In conclusion, *Lactobacillus* and *Saccharomyces* were the predominant bacterial and fungal genera, respectively. They showed a positive correlation with various flavours during light-flavour *Baijiu* fermentation. The uniformity of microbiota structure kept the stability of *Baijiu*. The differences between LP and YP fermentation could be due to *L. acetotolerans*, which mainly comes from the air and surface of the jar. Lack of *L. acetotolerans* on the surface of the jar might cause a lag of fermentation and lower ester content. As an important source of microorganism, the different environmental microbiota chartered the flavour of *Baijiu* in different periods. It can be concluded that the quality of *Baijiu* depends on well-balanced microbiota from the environment and starter. Exploring microbial ecology of *Baijiu* fermentation environments and processes could provide valuable information for understanding the complete ecology of *Baijiu* fermentation systems and can lead to enlightened perspectives for quality control.

## Methods

### Sample collection

To survey the effect of environmental microbiota on the fermentation of light-flavour *Baijiu*, samples were taken from Shanxi Xinghuacun *Fenjiu* Distillery Co. Ltd. (Fenyang, China) in 2016. The indoor air of the material preparing room and the inner surface of the fermentation jar were sampled as the critical environmental microorganism sources with long contacting time during the fermentation process. Samples at critical points in the fermentation process were taken from two different periods (LP in September and YP in December). According to the dynamics of physiochemical characters and microbial communities during fermentation^15^, we sampled the following critical points: day 0, 3, 7, 15, and 28. At each of the sampling point, approximately 300 g of fermented grains from the center of fermentation jars were collected in triplicate according to online sampling procedure and relative research^15^.

For each period, three batches were taken separately from different plants as shown in Table S1. Air samples were collected by an AirPort MD8 sampler (Sartorius, Goettingen, Germany) around the soaking and cooling place of the plant^39^. The air sampler was loaded with an 80 mm, sterile, gelatin membrane filter with a pore size of 3 μm and was fixed at a height of 1.5 m above the floor. A sample of 1000 L of indoor air was collected with a flow rate 50 L/min for 20 mins. After collection, the filter was removed to 5 mL sterile phosphate buffered saline (PBS). For each plant, a total of 3000 L of air were sampled from three places around the material preparation. A swab sample of the earthen jar was taken after routine cleaning and before the start of production. Surfaces of the jar were sampled with sterile cotton-tipped swabs that were moistened with sterile PBS and streaked across the target sampling site (100 cm^2^); overlapping S strokes with rotations of the swab were performed to ensure full contact of the swab tip with the surface. The swabs were placed into 5 mL of sterile PBS, stored on ice, and transported to the lab for testing. Mix the environment samples from same plant of same batch for microbial analysis.

### Physicochemical analysis

The pH was measured with a pH meter (Sartorius, Germany) inserted directly into the sample suspension (1 g/10 mL). The acidity of the samples was determined by titration with a standard 0.1 M NaOH solution. Moisture content of fermented grains was determined using a drying method at 105 °C until a constant weight was reached. Core temperature during fermentation was recorded with electronic temperature sensors (iButton, Maxim, USA) that were inserted into the centre of the fermented jars.

### Volatile compounds

Volatile compounds in fermented grains were assayed by headspace solid-phase micro extraction coupled with gas chromatography-mass spectrometry (HS-SPME-GC-MS). The sample (2 g) was mixed with Milli-Q water (8 mL) in triplicate, after ultrasonic treatment for 30 min, the sample solution was centrifuged at 8,000 × *g* at 4 °C for 10 min. The supernatant (8 mL; collected from triplicate samples), the internal standard 2 μL(4-methyl-2-pentanol, 125.0 mg/L), and sodium chloride (3 g) were placed into a 20 mL vial. The volatile compounds were sampled with an SPME fibre (50:30 mm divinylbenzene–carboxen–polydimethylsiloxane, DVB/CAR/PDMS; Supelco Co., Bellefonte, PA, USA) at 50 °C for 5 min and extracted for 45 min^40^. After extraction, the DVB/CAR/PDMS coated fibre was inserted into the injection port of the gas chromatograph for volatile compound analysis^41^. The compounds were identified by comparison with mass spectra data of the NIST 14 mass spectral database. The content of each compound was calculated by comparison of its area with the internal standard, 4-methyl-2-pentanol. The amounts of individual constituents present in the sample were calculated and expressed as micrograms per gram of sample.

### Microbiological analysis - culture dependent methods

Microbial counts were determined as described by Zheng, *et al*.^20^. Total aerobic bacteria were enumerated on Plate Count Agar (Oxoid CM035) and incubated at 30 °C for 48 h. LAB were enumerated on MRSA (Oxoid CM0361) with 0.1% (w/v) natamycin (Delvocid, DSM, Delft, The Netherlands) to prevent fungal growth. Plates were incubated at 30 °C for 72 h. Yeasts and moulds were enumerated on Rose Bengal Chloramphenicol Agar (Oxoid CM0549) that were incubated at 28 °C for 72 h.

### DNA extraction

DNA extraction was carried out with a Powersoil DNA Isolation kit. The swab and air samples were centrifuged at 10000 × *g* for 10 mins and resuspended in the buffer of the Powersoil DNA Isolation kit (MoBio, Carlsbad, USA), following the manufacturer’s instructions. DNA extraction of fermented grains was performed following manufacturer’s instructions with minimal modifications, 200 μl of bead solution was removed from the tube and 200 μl of PCI (phenol: chloroform: isoamyl alcohol) was added. The concentration and purity of extracted DNA were assessed by Nanodrop 2000 (Thermo Fisher Scientific, Wilmington, USA).

### 16 S/ITS Amplicon analysis

For bacteria, the V3-V4 domains of the 16 S rRNA genes were amplified using primers 338 F (3′-ACTCCTACGGGAGGCAGCA-5′) and 806 R (3′-GGACTACHVGGGTWTCTAAT-5′). For fungi, the internal transcribed spacer ITS2 regions were amplified with primers ZIT_F (3′-GCATCGATGAAGAACGCAGC-5′) and ZITS_R (3′-TCCTCCGCTTATTGATATGC-5′)^42^. Reaction conditions consisted of an initial 95 °C for 3 min followed by 35 cycles of 95 °C for 30 s, 55 °C for 30 s, and 72 °C for 45 s, and a final extension of 72 °C for 10 min. PCR was performed using a GeneAmp PCR system 9700 (Applied Biosystems, USA). The sizes and quantities of the PCR products were determined using 2% (wt/vol) agarose gel electrophoresis. PCR products were analysed by electrophoresis and then stored at −20 °C for future experiments. Amplicons were submitted to the Majorbio Bio-Pharm Technology Co., Ltd. (Shanghai, China) for Illumina paired-end library preparation, cluster generation, and 300-bp paired-end sequencing on a MiSeq instrument. Raw reads were de-multiplexed, quality-filtered, and analysed using QIIME (v.1.17). The representative bacterial OTU sequences were annotated using the Silva 123/16 S rRNA database by a QIIME-based wrapper of RDP-classifier (v.2.2). The fungal ITS database was clustered using the USEARCH (version 7.1) and aligned by the BLAST algorithm (version August, 2016)^43^. For both bacteria and fungi, a 97% identity threshold was set. Representative sequences for OTUs of *Lactobacillus* and *Pediococcus* during the fermentation process were double checked with the BLAST search program to classify them at the species level. Sequences with identity scores greater than 97% were resolved at the species level^26,44^. The identities of all hits were greater than 98%. Identification at the species level will be considered putative for the purposes of this specific study. Sequence analysis is available through the Sequence Read Archive database under accession number SRP131059. The representative sequences of each OTU were uploaded to GenBank under accession number MG857859 - MG859237, MG872076 - MG872311.

### Data analysis

A One-Way ANOVA with SPSS V22.0 (IBM, U.S.A) was used to determine the significance of different microbial enumeration data and physicochemical parameters. Principal coordinate analysis (PCoA) based on Bray-Curtis distance and analysis of Similarity (ANOSIM) were conducted to investigate the microbiota cluster by R software (v 3.2.2) with vegan packages. PCoA was performed with R function ‘cmdscale’. ANOSIM was performed with R function ‘anosim’ to check the significance of clusters revived from PCoA results. The multivariate statistical analyses on flavour were performed with the SIMCA-14.1 software (Umetricus, Sweden). For hierarchical cluster analysis (HCA), the distance between observations was calculated using *Ward’s* method based on the concentration of volatile flavours. Partial least square discriminate analysis (PLS-DA) was chosen to summarize volatile flavour data by grouping variables^45^. LP and YP membership was provided prior to the PLS-DA model and that group colours were added to PLS-DA plot according to the clusters retrieved from the HCA. Two-way Orthogonal PLS (O2PLS) was carried out by integration of microbiota (*X*) and flavours (*Y*) during alcoholic fermentation with SIMCA-14.1. In order to assess the quality and validity of models, the 7-fold cross validation and response permutation testing (n = 200) were performed. The correlation matrix shows the pair-wise correlation between all variables (X and Y). Terms with large variable importance in the projection (VIP), larger than 1, are the most relevant for explaining *Y*^46^. And pearson pairwise correlations between microbiota and flavours were calculated simultaneously using ‘corr.test’ function with psych package in R to analyze the significance of the correlation. Highly correlation coefficient (|*ρ*| ≥ 0.7) with *p*-value < 0.01 between microbiota and flavours was visualized via Cytoscape (v.3.4.0).

### Data availability

All data generated during this study are included in this published article (and its Supplementary materials). Sequence analysis is available through the Sequence Read Archive database under accession number SRP131059.

## Electronic supplementary material

Supplemental materials

Dataset 1
