# Supplementary material for: Effect of the environment microbiota on the flavour of light-flavour Baijiu during spontaneous fermentation
Source: Sci Rep. 2018 Feb 21;8:3396. doi: 10.1038/s41598-018-21814-y (PMC5821866; doi:10.1038/s41598-018-21814-y)

**Supplemental Information for**

# Effect of the environment microbiota on the flavour of light-flavour *Baijiu* during spontaneous fermentation


**Xiao-Na Pang[1, 2]; Bei-Zhong Han[1]; Xiao-Ning Huang [1]; Xin Zhang [3]; Lin-Feng Hou[1]; Ming Cao[1]; Li-Juan Gao[4]; Guang-Hui Hu[4]; and Jing-Yu Chen [1, 2, *]**

[1] *Beijing Laboratory of Food Quality and Safety, College of Food Science and Nutritional Engineering, China Agricultural University, Beijing 100083, China*

[2] *Beijing Advanced Innovation Center for Food Nutrition and Human Health, China Agricultural University, Beijing 100083, China*

[3] *Technology Center, Shanxi Xinghuacun Fenjiu Distillery Co. Ltd., Fenyang 032205, China*

[4] *Department of Biotechnology, Beijing Center for Physical and Chemical Analysis, Beijing 100089, China*

***Corresponding Author:** Jing-Yu Chen

P.O. Box 398, 17 Qinghua East Rd., Beijing 100083, China

Email: chenjy@cau.edu.cn

Phone: +86-10-62737966; Fax: +86-10-62737078

**This file includes:**

Supplementary tables (Table S1-S2)

Supplementary figures (Figure S1-S2)

**Other supplementary information for this manuscript includes the following:**

Supplementary dataset 1 as an Excel file: Supplementary_Dataset_S1.xls

# Supplementary table legends

Table S1. Sequencing information.

## Supplemental tables

Table S1. Sequence information.

| Sample code | Bacteria | | | Fungi | | |
|---|---|---|---|---|---|---|
| | Sequences | Bases (bp) | Average Length (bp) | Sequences | Bases (bp) | Average Length (bp) |
| LP_1_A | 42686 | 18000000 | 432.22 | 43635 | 14343343 | 328.71 |
| LP_2_A | 43950 | 19000000 | 435.26 | 43201 | 14692357 | 340.09 |
| LP_3_A | 31822 | 14000000 | 436.69 | 38260 | 13341221 | 348.7 |
| YP_1_A | 38514 | 17000000 | 437.71 | 44168 | 15400699 | 348.68 |
| YP_2_A | 44568 | 20000000 | 440.5 | 30961 | 10872293 | 351.16 |
| YP_3_A | 30053 | 13000000 | 438.66 | 34291 | 12042531 | 351.19 |
| LP_1_J | 36450 | 16000000 | 449.83 | 36372 | 12473667 | 342.95 |
| LP_2_J | 39634 | 18000000 | 449.79 | 35561 | 12670180 | 356.29 |
| LP_3_J | 32530 | 15000000 | 449.79 | 32868 | 11422392 | 347.52 |
| YP_1_J | 39988 | 18000000 | 441.8 | 44953 | 16311394 | 362.85 |
| YP_2_J | 33384 | 15000000 | 446.69 | 31980 | 11256577 | 351.99 |
| YP_3_J | 39387 | 18000000 | 448.22 | 43380 | 15025860 | 346.38 |
| LP_1_D | 43741 | 19000000 | 432.32 | 35777 | 12471215 | 348.58 |
| LP_2_D | 43038 | 19000000 | 445.95 | 35830 | 12546529 | 350.17 |
| LP_3_D | 33058 | 14000000 | 436.49 | 39893 | 14022739 | 351.51 |
| YP_1_D | 31970 | 14000000 | 444.5 | 37446 | 13093003 | 349.65 |
| YP_2_D | 33584 | 15000000 | 442.65 | 33215 | 11607498 | 349.47 |
| YP_3_D | 30403 | 14000000 | 447.52 | 36711 | 12315214 | 335.46 |
| LP_1_F0 | 39704 | 18000000 | 442 | 40195 | 14183965 | 352.88 |
| LP_2_F0 | 34027 | 15000000 | 438.7 | 39667 | 13964099 | 352.03 |
| LP_3_F0 | 35820 | 16000000 | 439.88 | 32359 | 11490426 | 355.09 |
| YP_1_F0 | 40063 | 18000000 | 441.56 | 42504 | 14858052 | 349.57 |
| YP_2_F0 | 35922 | 16000000 | 441.48 | 40026 | 14047574 | 350.96 |
| YP_3_F0 | 36726 | 16000000 | 443.87 | 41457 | 14612057 | 352.46 |
| LP_1_F3 | 32498 | 14000000 | 442.17 | 37782 | 13596773 | 359.87 |
| LP_2_F3 | 35434 | 16000000 | 442.98 | 35714 | 12777818 | 357.78 |
| LP_3_F3 | 43761 | 19000000 | 444.34 | 44713 | 16075550 | 359.53 |
| YP_1_F3 | 33319 | 15000000 | 440.33 | 42569 | 15334370 | 360.22 |
| YP_2_F3 | 32084 | 14000000 | 443.79 | 39436 | 14259085 | 361.58 |
| YP_3_F3 | 44145 | 20000000 | 449.56 | 41068 | 15527661 | 378.1 |
| LP_1_F7 | 37532 | 17000000 | 449.73 | 31475 | 12481489 | 396.55 |
| LP_2_F7 | 42731 | 19000000 | 447.12 | 44108 | 17011799 | 385.69 |
| LP_3_F7 | 42029 | 19000000 | 449.9 | 41611 | 16186249 | 388.99 |

| | | | | | | |
|---|---|---|---|---|---|---|
| YP_1_F7 | 43798 | 20000000 | 449.41 | 38737 | 14991886 | 387.02 |
| YP_2_F7 | 40279 | 18000000 | 449.35 | 31261 | 12156030 | 388.86 |
| YP_3_F7 | 41314 | 19000000 | 449.47 | 39197 | 15015467 | 383.08 |
| LP_1_F15 | 37650 | 17000000 | 448.98 | 35919 | 14173877 | 394.61 |
| LP_2_F15 | 43039 | 19000000 | 449.81 | 35338 | 14054228 | 397.71 |
| LP_3_F15 | 37492 | 17000000 | 449.8 | 44578 | 17009656 | 381.57 |
| YP_1_F15 | 41241 | 19000000 | 449.89 | 42917 | 16584049 | 386.42 |
| YP_2_F15 | 42166 | 19000000 | 449.84 | 37904 | 14663517 | 386.86 |
| YP_3_F15 | 40367 | 18000000 | 449.94 | 37971 | 14543582 | 383.02 |
| LP_1_F28 | 42352 | 19000000 | 449.6 | 35017 | 13427036 | 383.44 |
| LP_2_F28 | 41752 | 19000000 | 449.74 | 39732 | 15794072 | 397.52 |
| LP_3_F28 | 34390 | 15000000 | 449.83 | 43492 | 16716813 | 384.37 |
| YP_1_F28 | 31525 | 14000000 | 449.9 | 37191 | 14717833 | 395.74 |
| YP_2_F28 | 30062 | 14000000 | 449.94 | 43215 | 16425461 | 380.09 |
| YP_3_F28 | 42912 | 19000000 | 449.96 | 37945 | 14638117 | 385.77 |

Comments: LP: *Lipai* period means the beginning of a production cycle in September after summer production break. YP: *Yuanpai* period which refers to the period between LP and *Tiaopai* (last production period). The numbers in the middle of sample code represent the code for plant 1, 2, and 3 respectively. A: Air. J: Surface of the jar. D: *Daqu*. F followed by number means fermentation time.

Table S2. ANOSIM testing the effect of different sampling time on the microbial community structure based on Bray-Curtis distance index.

| Microbial community | Source of variation/samples/effect | R Statistic | *P* value |
|---|---|---|---|
| Bacteria | *Daqu*, Air and surface of jars | 0.8799 | 0.001 |
| | Fermented grains of different time | 0.6089 | 0.001 |
| Fungi | *Daqu*, Air and surface of jars | 0.7849 | 0.001 |
| | Fermented grains of different time | 0.898 | 0.001 |

Comments: *P*-value <0.05 indicates significant difference between groups. A large and positive R statistic indicates dissimilarity between groups.

Figure S1. HCA analysis of volatile flavours during light-flavour Baijiu fermentation in different periods.

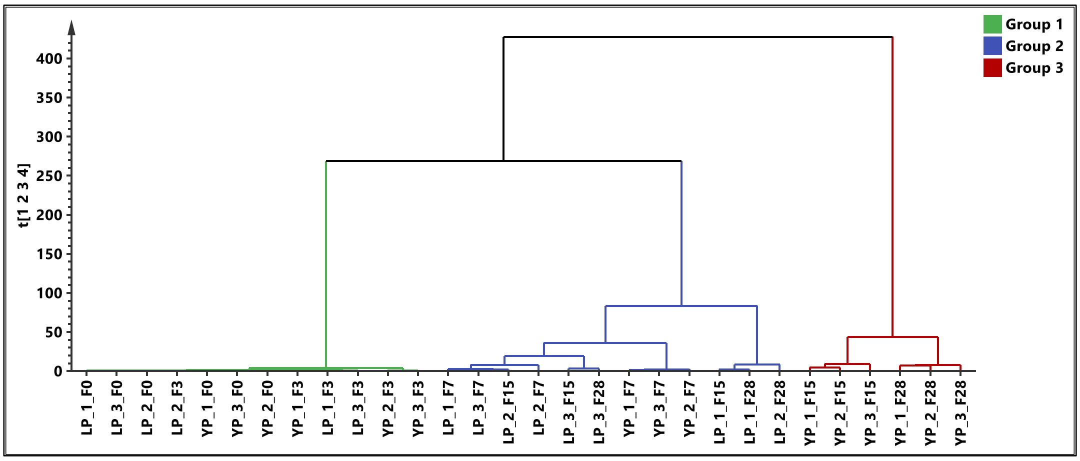

Figure S2. Permutation tests (n = 200) performed by SIMCA. (a) Permutation test based on O2PLS model between microbiota and flavor. (b) Permutation test on O2PLS model between LAB and flavor. The criteria for validity are: All blue $Q^2$-values to the left are lower than the original points to the right. Or the blue regression line of the $Q^2$-points intersects the vertical axis (on the left) at, or below zero.

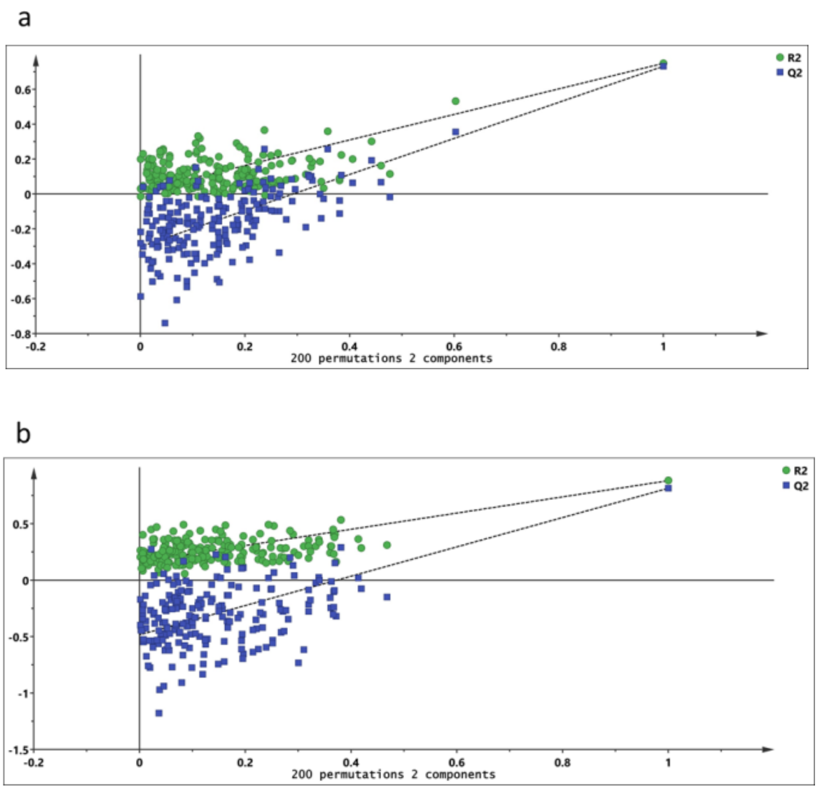

Supplement: Supplementary file 1 — Supplemental materials [file 41598_2018_21814_MOESM1_ESM.pdf]
